# Tensile Performance of Headed Anchors in Steel Fiber Reinforced and Conventional Concrete in Uncracked and Cracked State

**DOI:** 10.3390/ma15051886

**Published:** 2022-03-03

**Authors:** Panagiotis Spyridis, Nikolaos Mellios

**Affiliations:** Faculty of Architecture and Civil Engineering, TU Dortmund University, 44227 Dortmund, Germany; connections.bauwesen@tu-dortmund.de

**Keywords:** steel fiber reinforcement, fiber reinforced concrete, fastening technology, anchorage to concrete, concrete breakout failure, concrete cracking

## Abstract

Steel fiber reinforced concrete (SFRC) is currently the material of choice for a broad range of structural components. Through the use of SFRC, the entire, or a large portion of, conventional rebar reinforcement can be replaced, in order to improve the load-bearing behavior but also the serviceability and durability characteristics of engineering structures. The use of fiber reinforcement therefore plays a vital role in acute current and future construction industry objectives, these being a simultaneous increase in the service life of structures and the reduction of their environmental impact, in addition to resilience to extreme loads and environmental actions. Next to the extended use of SFRC, modern construction relies heavily on structural connections and assembly technologies, typically by use of bolt-type cast-in and post-installed concrete anchors. This paper addresses the influence of fiber reinforcement on the structural performance of such anchors in SFRC and, particularly, the load bearing behavior of single headed anchors under axial static loads in uncracked and cracked concrete. Along with a presentation of background information on previous studies of SFRC with a focus on anchor concrete breakout failure, the experimental investigations are described, and their results are presented and elaborated on by consideration of various research parameters. A comparison with current design approaches is also provided. The conclusions are deemed useful for structural engineering research and practice.

## 1. Introduction

### 1.1. General

Steel fiber reinforced concrete (SFRC) is currently the material of choice for a broad range of structures such as industrial floors (e.g., in manufacturing or processing plants and storage facilities), prefabricated elements, thin shells, segmental and sprayed as well as cast final tunnel linings, special foundations and slabs on grade, watertight and containment structures (immersed structures, silos, nuclear facilities), and protection and defense structures. Furthermore, its use is also confirmed for many commonplace concrete component design situations, replacing the entire or a large portion of conventional rebar reinforcement in order to improve their load-bearing behavior as well as their serviceability and durability characteristics [1,2,3,4,5,6,7,8,9,10,11]. The use of fiber reinforcement thus plays a vital role in acute current and future construction industry objectives, these being a simultaneous increase in the service life of structures (by limitation of failure probabilities and improved durability) and the reduction of their environmental impact (reduced use of cement and structural steel and thinner cross sections), in addition to their resilience to extreme loads and environmental actions [12,13,14].

The main function of the fiber reinforcement is to improve the concrete crack formation and propagation, which is mostly related to an increased tensile strength and ductility of the material. Other basic material properties of fiber concrete, such as compressive strength or Young’s modulus, are only marginally influenced. In current practice, fibers used to improve the structural resistance of concrete are typically made of steel, where both the peak and residual tensile retention at various crack openings can be improved. This implies that tensile stresses and crack propagation are arrested by the fibers locally until (i) the fiber itself ruptures, (ii) the fiber is pulled-out from the concrete matrix, or (iii) the crack develops solely in the matrix between the distributed fibers. It is thus important to highlight that this overall response of SFRC is strongly influenced by the fiber dosage, but also by the fiber material and bond strength. The bond strength of the fiber depends, in turn, on its shape and end form and the matrix strength properties (see also [15,16] for more in-depth information). Understandably, the orientation of the fibers in the concrete matrix may also be decisive as regards the relative stress/resistance stress vectors (isotropic or rather orthotropic behavior) [17]. In all cases, the bond performance of fibers and their rupture strength governs their efficiency at the local level of crack intersections (this can, e.g., be identified by fiber pull-out tests), whereas their length, dosage, and distribution within the matrix are additional characteristics for the macroscopic material behavior (identified by means of material or component testing).

Next to the extended use of SFRC, modern construction relies heavily on structural connection and assembly technologies. A prominent sector in this field is the concrete fastening technology, which deals with connections to concrete substrates by use of bolt-type cast-in and post-installed anchors. Depending on the anchorage mechanism, the load direction, and the dimension and material strength properties of the anchors involved, different failure modes can be observed. Focusing herein on quasi-static actions, possible failure modes for tension loaded anchors may be briefly described as follows: steel tensile failure, pull-out of the anchor, concrete cone failure (in excess of the concrete tensile bearing capacity), and concrete splitting or side blow out of an adjacent free edge (due to transverse radial stresses around the anchor). Respectively, in shear, anchors may fail under steel rupture of the anchor (eventually with flexural bending for stand-off applications), pry-out failure (due to generation of a fracture surface on the counter-loading direction as a consequence of levering response of the anchor rod), and concrete edge failure or breakout, particularly for anchorages close to a free edge of the concrete. A combination of the above failure modes is also possible. For a detailed description of the failure modes, see [18]. The ultimate limit state design of anchorages according to current standards relies on the evaluation of the most critical load resistance from each of the above failure modes [19]. Concrete related failure modes are of particular interest for two main reasons: (a) they are, in principle, quasi-brittle, and as such, they may develop without preceding signs of damage, so there is limited possibility for a ductile response of the steel components for remediation action and avoidance of complete anchorage system failure, and (b) concrete failure leads to extensive damage in the area of the fastening, and there is limited possibility for adequate repair. This, consequently, leads to a particular interest in the adequate assessment of concrete cone failure resistance under tension, which also serves as a reference value in the realm of fastening technology and is the main interest of this study. Furthermore, it is also well known that cracks in the concrete substrate are often present. These discontinuities in the base material of an anchor can significantly affect the stress distribution associated with the localized load transfer. As a result, the concrete related failure modes and their respective resistances are very sensitive to the existence of cracks in the concrete. 

### 1.2. Failure Load Prediction and Design in Unreinforced Concrete

To estimate the mean capacity of a single headed anchor in plain uncracked concrete without edge influence, the well-established Concrete Capacity Design (CCD) method according to 18 can be used, as expressed in Equation (1), where $N_{Ru,c,uncracked}^{}$ is the mean uncracked capacity, 16.8 is a calibration factor specific for the anchor type or product, $h_{ef}$ is the anchor embedment depth (the distance of the failure invitation point to the free surface), and $f_{c}$ is the mean cylinder compressive strength. For the case of a headed stud anchor located in a plane crack with a width of 0.3 mm, Eligehausen et al. [20] estimates a mean breakout strength at slightly above 75% of the reference value per Equation (1). Indicatively, this mean value further reduces by 73% and 68% for a crack width of 0.5 and 1.0 mm, respectively. For design purposes, the European Committee for Standardization [19] proposes the calculation of the characteristic resistance through Equation (2), where $N_{Rk,c}^{}$ stands for the characteristic resistance and $f_{ck}$ is the characteristic cylinder compressive strength, and it recommends *k* = $k_{ucr}=12.7$ and $k_{cr}$ = 8.9 (equal to 0.7 · $k_{ucr}$) for cast-in headed studs in uncracked concrete and concrete with a 0.3 mm crack, respectively.

(1) $$N_{Ru,c,uncracked}^{}=16.8\cdot h_{ef}{}^{1.5}\cdot \sqrt{f_{c}}\;[N],$$

(2) $$N_{Rk,c}^{}=k\cdot h_{ef}{}^{1.5}\cdot \sqrt{f_{ck}}\;[N]$$

### 1.3. Previous Research on Fastenings in SFRC

In probably the first ever investigation on anchors in SFRC, Holschemacher et al. [21] reports axial tests on expansion, undercut, and bonded anchors, with waved sheet cut and hook-ended steel fibers with a length/diameter characteristic ratio (L/D) of 35/1.2 and 50/0.8, respectively, steel material strength below 1050 MPa, and a dosage of 50 kg/m^3^. The mean concrete cylinder compressive strength ($f_{c}$) was 28 MPa. Expansion and undercut anchors failed with cone breakout for normal concrete and pull-out in SFRC, while bonded anchors failed with steel failure or pull-out regardless of fibers. An overall negligible difference in mean values has been shown, while anchorages in SFRC showed a much higher scatter, which was attributed to the higher porosity of SFRC, the inhomogeneous distribution of fibers in the mix, and the inhomogeneous fiber orientations. Gesoglu et al. [22] carried out tension tests on M12 and M16 epoxy bonded and grouted threaded rods at a $h_{ef}$ ranging from 40 to 160 mm. The concrete mixes used had an *fc* of 30 and 50 MPa, without fibers or with steel fibers of L/D of 60/0.8 mm at dosages of 60 to 80 kg/m^3^, respectively. The fiber strength is not noted, but due to the date of the paper and the available products in the market at the time, this was likely in the range of 1000 MPa. The anchors failed by combined pull-out and pure concrete cone failure, while the use of steel fibers in concrete did not significantly affect the pull-out capacity of the anchors, but it increased overall ductility and the failure type of some anchors shifted from concrete cone failure to pull-out mode or combined pull-out and cone mode. This implies some increase in concrete cone failure resistance. Similar results are proposed by Kurz et al. [23], which tested various anchor M10 types with *h_ef_* between 65 and 75 mm in concrete with $f_{c}$ in the range of 50 MPa, round/crushed gravel aggregate and dosage of 25 and 60 kg/m^3^ of hooked-ended fibers with L/D = 60/0.75 mm, and material strength 1255 MPa. In particular, this study shows a 15% increase in concrete cone resistance in fiber concrete, but with a large scatter that does not lead to an increase at characteristic level. Coventry et al. [24] tested M10 chemically bonded anchors at *h_ef_* = 90 mm in concrete with compressive strength in the range of 45 MPa, reinforced with hooked ended fibers of L/D = 50/1.0 mm and material strength 1050 MPa at dosages of 20 kg/m^3^, 40 kg/m^3^, and 60 kg/m^3^. The tests report concrete cone failure with an increase in failure load of 5.7, 8.6, and 11.4% in respect to the before mentioned fiber dosages. Grosser [25] reports experimental results on shear related concrete edge failure of M16 anchors with a *h_ef_* = 100 mm and edge distances of c_1_ = 50–100–150 mm, in normal strength concrete with a 25–35 kg/m^3^ content of hooked ended fibers of L/D = 50/1.0 mm and material strength 1100 MPa. These tests showed that the concrete breakout strength can be increased by more than 20% in SFRC, and particularly for small edge distances (up to 50% increase). Cattaneo et al. [26] carried out tension and shear tests on expansion anchors at various embedment depths in high strength concrete of 100 and 120 MPa compressive strength with hook-ended steel fibers of L/D = 20/0.38 at 50 and 70 kg/m^3^, respectively. The tests indicated an increase in concrete edge failure resistance under shear of up to 98%, and a mild to negligible increase in tensile and inclined tests. The same research group in [27] presents unconfined tests of M12 epoxy bonded anchors with *h_ef_* of 50, 75, and 100 mm in concrete with *f_c_* of 30, 75, and 90 MPa, without fibers and with steel hooked-ended fiber dosages of 50 and 70 kg/m^3^. The fibers had an L/D of 30/0.38 mm of unreported strength (but presumably >3000 MPa). Tests in SFRC with smaller embedment showed an increase in cone breakout resistance in the range of 10 to 20%, while a transition to steel and pull-out failure of the anchor also indicated a relatively higher (non-governing) concrete cone failure load for fiber concrete. Nilforoush et al. [28] presents tests on an M36 headed anchor with *h_ef_* = 220 mm in two concrete batches with strengths in the range from 40 to 80 MPa, including hooked-end fibers with L/D of 60/0.92 (of unreported strength) and a dosage of 80 kg/m^3^. All tests led to concrete cone failure, while some tests without fibers also exhibited splitting, and tests with fibers exhibited radial cracking. The tests showed an increase at failure load of 25% for normal and 40% for high strength concrete. Dengg et al. [29] tested various M8 to M12 anchor systems of various types (undercut, drop-in, concrete screw, expansion, bonded, plastic anchors), with *h_ef_* between 40 and 70 mm, in special concrete mixes of compressive strength below 30 MPa with recycled, schist aggregate from tunnel excavation. The mixes included 30 kg/m^3^ of hooked-end steel fibers of L/D = 50/0.75 mm and material strength 1450 MPa with or without additional polypropylene fibers, yet this did not significantly influence the resistance against concrete cone failure. Hlavicka et al. [30] reports pull-out tests of vinyl ester and epoxy bonded anchors in concrete with a strength ranging between 35 and 55 MPa, including mixes with hooked ended steel fibers with L/D of 50/1.0 mm and strength of approx. 1100 MPa, and smooth fibers with L/D of 12/0.2 mm and strength of 3000 MPa, and it shows a steady increase, with fiber content and strength of up to 44% (for 80 kg/m^3^). Delhomme et al. [31] also presents bonded anchors of *h_ef_* = 40 mm failing with combined bond–pull-out failure in concrete with a special ultra-high-performance concrete with an *fc* > 165 MPa and a hybrid steel fiber dosage of 160 kg/m^3^, i.e., with L/D of 13/0.1 mm + 20/0.3 mm. Although the material strength is not specified, it is presumably > 2000 MPa. For concrete related failure nodes, the tests do indicate increased load capacity and a transition of failure modes from concrete cone to pull-out or steel rupture modes due to increased tensile strength of the concrete substrate. Schwenn et al. [32] presents tests of concrete screws with *h_ef_* = 40 mm in concrete with a 150 MPa strength using fibers of L/D = 6/0.2 mm and a 2800 MPa strength, at 150 kg/m^3^ dosage. A comparison of their concrete cone resistance, with the existing predictive equation as per 18, indicates an expected resistance increase in the range of 40 to 55%. Tóth et al. [33] defines for the first time the geometric conditions that have to be fulfilled for the steel fibers to have a beneficial effect on the concrete break out. It comprises pull-out tests for groups and single anchors in concrete of strength in the range of 53 to 65 MPa and dosage of 30 and 50 kg/m^3^ hooked-end fibers with L/D of 35/0.75 and strength of 1200 MPa. The tests were carried out on M16 epoxy bonded threaded rods with τ = 35 MPa and *h_ef_* = 70 mm, and they included tension on single and triple groups and shear tests toward a free edge (with edge distances of c_1_ = 60 and 85 mm). Concrete cone and concrete edge failure for tension and shear tests, respectively, were observed, with a definite increase in capacity compared to plain concrete. The improvement in the displacement behavior of the anchors that leads to an enhanced performance was highlighted. It was shown that a better re-distribution of anchor forces within an anchor group leads to an even higher group resistance than that of single anchors in a corresponding concrete mix. The ultimate tension load for single anchors was found to be increased by 50% for a 30 kg/m^3^ dosage, but only by 30% for a 50 kg/m^3^ dosage. This study also closely addressed the aspect of fiber orientation homogeneity, since the casting boundaries lead to a de-facto parallel alignment of the fibers close to the formwork and free surfaces of the specimen. In particular, this layer of non-homogeneous fiber orientation was proposed to be 1.7 times the fiber length. Ayoubi et al. [34] presents tests on anchor channels, with cast-in headed bolts in various embedment depths, in concrete with a 95 MPa strength and a fiber dosage of 40 kg/m^3^, concluding with a clear increase in concrete breakout capacity (up to approx. 20%) and, consequently, also a transition from breakout in plain concrete to fastening steel failure in SFRC. Vita et al. [35] presents tension tests on M16 and M24 epoxy resin bonded anchors with embedment depths at 65 and 100 mm in concrete with strength in the range of 35 and 60 MPa, and with inclusion of hooked ended steel fibers with L/D of 35/0.55, material strength of 1200 MPa, and dosages of 40 and 80 kg/m^3^, failing exclusively by cone breakout. The tests mainly focused on the influence of 0.3 mm cracks on the overall performance of the anchors, but from reference tests on uncracked concrete, it is seen that an approx. 12% increase in ultimate loads was observed for a dosage uptake from 40 to 80 kg/m^3^. Tests by Kocur et al. [36] on M16 and M20 crimped sleeve anchors with varying embedment depths in normal strength concrete, with hooked ended fibers, with L/D of 35/0.55 and a 1550 MPa strength, also confirm a strong increase in concrete breakout resistance of approx. 30% and 45% for fiber dosages of 30 and 50 kg/m^3^, respectively. Of the studies described above, only [29,31,35] have dealt with cracking as an investigation parameter. Moreover, the first two of these studies focus on special concrete types, namely concrete with recycled aggregates and ultra-high-performance concrete with large crack widths (1 mm), respectively, whilst tests in [31] developed anchor pull-out instead of concrete cone failure. Dengg et al. [29] presents comparative tests of drop-in and concrete screw anchors in concrete with a 0.3 mm crack width and indicates that failure loads in cracked SFRC are less than 20% lower compared to the values for uncracked concrete. As mentioned above, 35 investigates specifically the concurrence of 0.3 mm cracks in SFRC, and it concludes that the ratio of concrete breakout mean resistance values in cracked to uncracked concrete are on average 0.81 and 0.67 for the lower and higher concrete strength ranges, respectively. A previous recent study by the authors focused on the influence of different fiber types on the breakout performance of anchorages solely in uncracked concrete [37]. Currently, there is no comparative study focusing on the load bearing capacity of headed anchors in SFRC between cracked and uncracked status and with respect to different fiber performance.

### 1.4. Current Design Proposals for Concrete Breakout Failure of Fastenings in SFRC

With respect to design and specification in concrete fastenings, currently, there is no coverage by product specification for use in SFRC, and this application finds its place either by use of project specific testing of anchors (design by testing) or by the simplifying assumption that fibers do not contribute to the strength of concrete breakout. Regarding design equations, Tóth et al. [33] based on tests from the literature as well as the authors’ own tests, proposes a formula that predicts the characteristic (lower bound) breakout resistance in uncracked concrete, based on own tests and tests from literature. Based on this proposal, Equation (2) is multiplied with the factor $\gamma_{fibre}$, acc to Equation (3), where $k_{fibre}$ is the dosage of fibers in kg/m^3^. The formula’s applicability is limited to anchors extending outside the non-homogeneous fiber orientation zone (1.7 times the fiber length) and for hooked-ended steel fibers with a dosage between 30 and 80 kg/m^3^. The applicability of the formula is extended by the same authors in [38] for fiber dosages down to 20 kg/m^3^ and for straight and corrugated steel fibers, with a minimum material strength of 1000 MPa and lengths between 35 and 60 mm, but always larger than three times the maximum aggregate diameter. Vita et al. [35] introduces a multiplier of 0.7 in order to extend the applicability of the design equation proposed in [33] for concrete with a crack width of 0.3 mm.

(3) $$\gamma_{fibre}=1+\frac{k_{fibre}}{300}\;\leq 1.25$$

### 1.5. Objective of This Article

To date, product specifications and design standards do not cover fastenings in SFRC. However, as presented above, strong research interest is drawn to this topic. While various significant research efforts have focused on normal and high-performance concrete with different anchor types and fiber characteristics individually, an insight into the influence of fibers with different characteristics under otherwise comparable conditions is currently scarce. This article aims to extend the results from [37] in this regard, based on extensive experimental investigations, for cast-in place headed anchors and varying reinforcement configurations including rebar reinforcement and various fiber types and dosages. The study particularly draws focus on different concrete crack states, with the aim of identifying the influence of different reinforcement types on the load resistance of the anchors. Based on the experimental investigations reported below, useful conclusions can be drawn for the overall performance as well as the practical design of fastenings in reinforced concrete and SFRC.

## 2. Materials and Methods

### 2.1. Overview of Test Program

**Figure 1:**
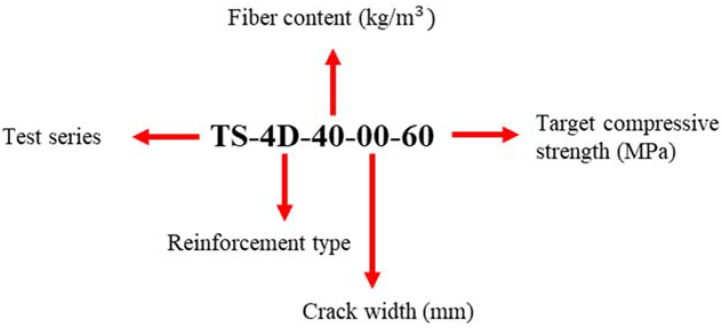
Graphical explanation of test annotation.

As seen in Table 1, the investigated parameters of the present study focus on different fiber/reinforcement type, fiber dosage, concrete strength, and crack width. The tests were carried out for cast-in headed studs with an embedment depth of 100 mm and the failure mode was consistently concrete cone breakout for all tests. The test annotation (see also Figure 1) includes parts following the letters TS (Test Series), namely defining the fiber/reinforcement type based on the product name (3D, 4D, 5D) that is discussed thoroughly in Section 2.2.2, the fiber dosage, crack width, and the targeted concrete strength. The yellow colour is used consistently in the graphs to denote tests in 3D fibers, blue for 4D fibers, red for 5D fibers, green for rebar reinforced (RB), and black for the reference tests in unreinforced/plain concrete. The combinations of concrete strength, fiber types, and fiber contents have been selected as such to represent commonplace recipes in practice. The rebar quantum represents a minimum reinforcement for crack control of the tested body, while the steel quantity nearly equals the maximum used steel fiber dosage (80 kg/m^3^). Both the tests and the concreting were carried out in the experimental hall of the TU Dortmund University (Institute for Building Research). 

### 2.2. Materials

#### 2.2.1. Concrete

For the concrete mix, CEM I 52.5 N Portland Cement and natural aggregates with a maximum size of 16 mm were used. The distribution for all concrete mixes was at 45, 20, and 25% for a sieve pass of 0/2, 2/8, and 8/16 mm, respectively. This composition is also in close agreement with the certification testing guide for mechanical anchors [39]. The overall mixes remained the same, with the differences of fiber addition, w/c ratio (between 0.40 and 0.60 for higher and lower strength classes, respectively), and the fact that higher strength concrete included superplasticizer based on highly concentrated polycarboxylate ether (PCE) with a dosage of 300 mL per 125 kg of cement to facilitate the workability.

#### 2.2.2. Fibers

**Figure 2:**
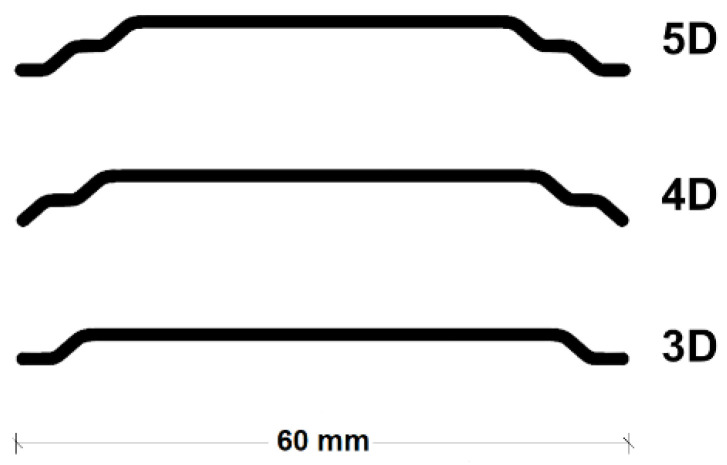
Shapes of fibers used.

The fibers used were of three main types of the product with the market name Dramix 3D 65/60BG, 4D 65/60BG and 5D 65/60BG, all by the same manufacturer, NV Bekaert SA, which are filaments of cold drawn wire with different hook-end shapes and material strength and deformability. Μain properties of the fibers used are shown in Table 2, while Figure 2 presents the actual shapes of the fibers. As seen in the different fiber types 3D, 4D, and 5D, the end anchorages possess more deflection points, a higher material strength, and greater elongation capacity. As a consequence, the fibers’ efficiency in crack bridging is increased both in terms of pull-out from the matrix as well as fiber rupture. More detailed descriptions of the fiber types’ differences and the associated overall structural performance can be found in [40,41]. For the respective test series, a mesh reinforcement was formed, with rebars every 100 mm, and diameter 10 mm only at the surface of the concrete specimen. 

#### 2.2.3. Anchors

**Figure 3:**
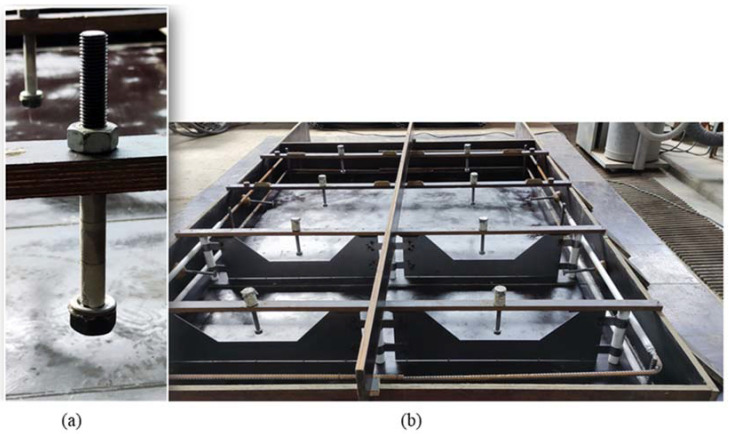
(**a**) Cast-in anchor prior to concreting; (**b**) Overview of formwork for concrete specimen with crack inducers (metal sheets), ring reinforcement, and positioning of anchors with protection cap.

The cast-in fastening elements were formed by means of M16 12.9 threaded rods with a passing HV hex-nut and round washer acc. To [42,43] at their bottom end, leading to an embedment depth of *h_ef_* = 100 mm. The anchor head was formed by screwing the hex nut and stabilizing the washer on top of it by hot glue, creating a head bearing external diameter of 30 mm. The rod was taped along its remaining embedment to prevent inrush of concrete and bonding along the threads (see Figure 3a).

#### 2.2.4. Material Testing

Properties of the concrete substrate’s material based on cored cylinder specimens with a height of 200 mm and a diameter of 95 mm (compressive and splitting tests), as well as concrete cubes with a 150-mm dimension, were also obtained, and they are presented herein as part of Table 3 for reference. Six cube specimens were cast simultaneously with the main concrete specimens, and then three were tested at the day of commencement of the anchor tests and the rest immediately after completion. Cored specimens were always taken in a direction parallel to the anchor axis, after the completion of the anchor tests at intact regions of the specimens. Three probes were used for compression and three for splitting tests. The age of the concrete at anchor and cube tests was on average 39 days and at cylinder testing 86 days.

### 2.3. Testing Configuration and Procedure

The cube and anchor tests were performed 28 to 30 days after concreting of the respective specimen, while cored samples were tested within 4 weeks thereafter. The testing dates are also shown in Table 1. The specimens had a width of 2000 mm, a height of 250 mm, and a length—depending on whether they host 8 or 12 anchors—of 2750 or 4050 mm. They were cast in wooden formwork with the anchors held by holed planks at the open surface of the formwork, and at a minimum distance of 3 × *h_ef_* (300 mm) from each other and the free edges, in order to allow for complete development of a possibly broader breakout body due to the fibers. The embedment depth and the minimum distances from free edges were in agreement with [33] to achieve an unbiased fiber orientation and homogeneity. In order to mitigate the influence strength inconsistencies that occur naturally between each concreting, typically, a concrete specimen contains both crack and uncracked configurations for anchor testing. The concrete mix including the fibers was performed simultaneously and the cast material was compacted by means of a hand-held vibrator. All concrete elements were smoothed and cured in a free internal area of the laboratory, covered by a foil.

Additional arrangements were required for the cracking of the concrete. Three PVC tubes were cast at each crack plane, perforating the concrete specimen, to host wedges applied to induce cracking. A trapezoidal metal sheet was added, crossing concentrically the location of the anchors. In addition, PVC tubes were used at crack intersections by rebars, to locally eliminate the reinforcement bond. The wedges were then hammered in place until the desired crack width was achieved in the entire length of the concrete specimen. Crack inducers assured that the cracks intersected concentrically with the anchors. The entire system was kept in place throughout the testing of the anchors to avoid the closure of the initial crack. During the testing, the crack might slightly increase only in the immediate area of the anchor, therefore, the crack was not measured continuously during testing.

**Figure 4:**
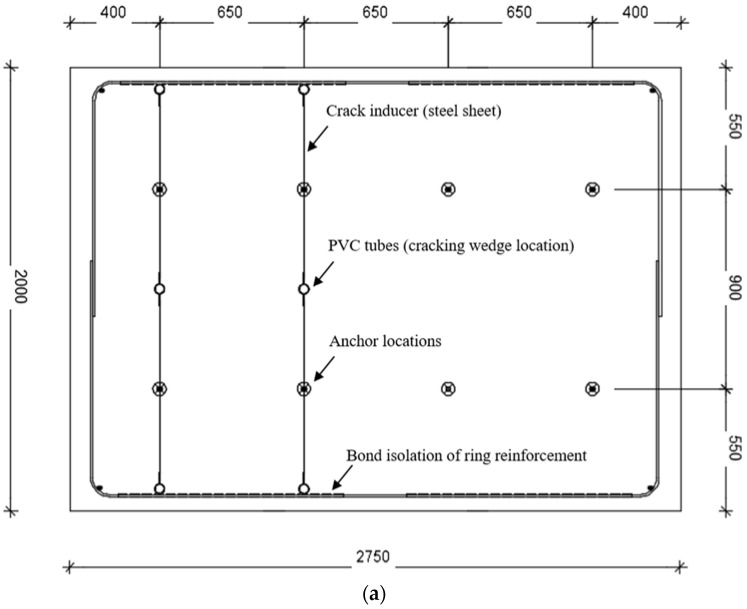

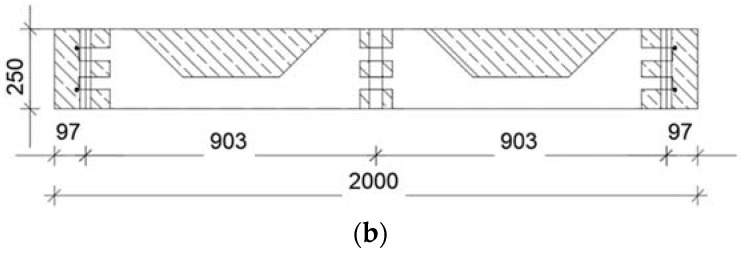
(**a**) Indicative concrete specimen layout for eight anchors test (four in uncracked and four in cracked concrete). Dimensions in mm. (**b**) Concrete specimen cross section view with a crack inducer layout. Dimensions in mm.

A drawing of the concrete specimen is given in Figure 4a. An overview of the actual molds and a detail of the anchor is given in Figure 3. The cross-section view of the specimen with the basic dimensions of the crack inducer is given in Figure 3b.

**Figure 5:**
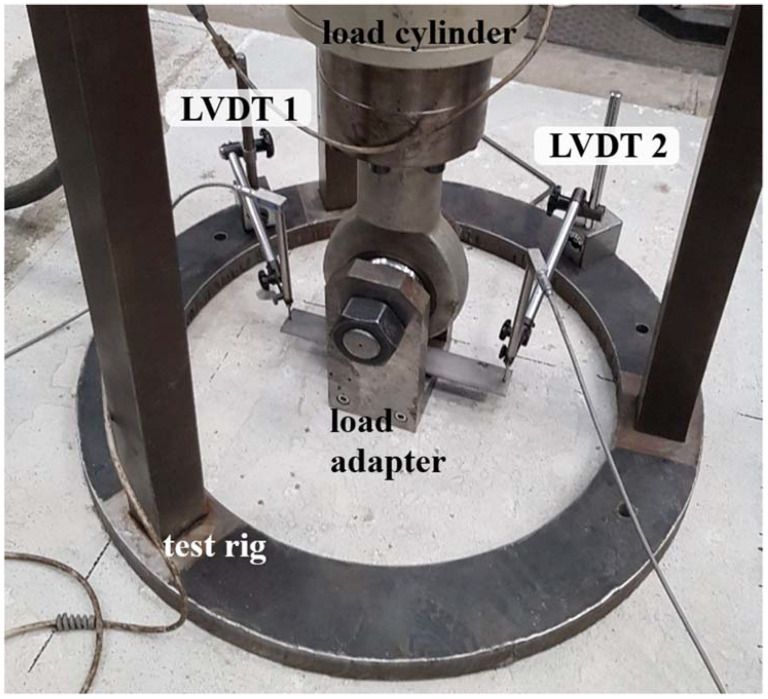
Test configuration, indicating the load cell and adapter, test rig with an internal support radius of 600 mm, and LVDT displacement transducers, fixed at the support ring and measuring close to the anchor head level.

In general, the tension tests on single bonded anchors were carried out based on the recommendations given in [38], and the overall setup can be seen in Figure 5. For the testing, a 250 kN servo-hydraulic cylinder with an integrated load cell was used, with a load direction centered and aligned to the anchor’s axis. A ring support of the test rig was used at diameter greater than 3·*h_ef_* from the anchor. In order to obtain the anchor displacement, two Linear Variable Displacement Transformers (LVDT) by Messotron GmbH & Co KG with market name WTH 20 l R/M, were placed at the pull-out fork adapter diametrically of the anchor in order to statistically process and reduce any displacement measurements errors. The load speed was set to 0.2 mm/min, and only after reaching 70% of the maximum load in the descending load-displacement branch, the speed would be increased to 1 mm/min. 

## 3. Results and Discussion

### 3.1. Overview of Results and Variability Evaluation

**Figure 6:**
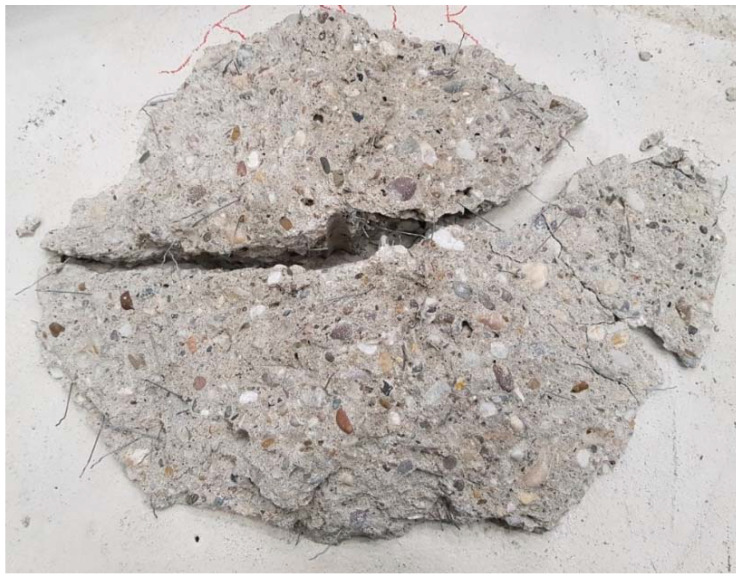
Representative concrete cone breakout body (bottom view).

The results of all the experiments in terms of ultimate loads are summarized in Table 3, alongside their average loads and coefficient of variations, but also main material tests results carried out for the concrete specimens. In total, 124 tests of anchors in tension were conducted; all tests failed by concrete cone breakout. A representative failure body can be seen in Figure 6. In order to keep tests performed in concrete with various compressive strengths comparable, every test result is normalized to a hypothetical cube strength of 45 MPa, by multiplication with the normalizing factor α = $\sqrt{45/f_{c}}$, where *fc* is the recorded cube compressive strength. 

The coefficient of variation in all anchors tested in uncracked concrete remains below 10%, except for one test series (TS-5D-25-00-60), which exhibits a coefficient of variation of 13.3%. In the case of anchors in cracked concrete, the coefficient of variation is higher but still remains in reasonable margins. The highest coefficient of variation was 20.2% in the test series TS–4D–40–03–60 in cracked concrete. The highest dispersions in the tests for uncracked concrete (variation coefficients of 0.133 and 0.094) appeared for the lowest tested fiber dosages (25 kg/m^3^), but the dispersions in cracked concrete do not seem to follow a distinct pattern depending on any of the investigation parameters. However, a relatively high scatter of results, namely 10.9%, was observed for TS–5D–25–05–60. This may suggest that the test series in higher strength concrete with a 5D fiber dosage of 25 kg/m^3^ is associated with a deviation from the overall pattern of the tests, possibly due to a concrete batch quality issue, yet this is not substantiated by a closer review of the test procedure and the statistical interpretation of the results herein. Further, all test series have been subjected to the Grubb’s test and no outlier has been identified within each probe set of four tests. The coefficients of variation for the material tests were up to 11% 13% for cube and cylinder compression testing, respectively. It should be mentioned that the compressive strength of both types specimen is in the same order due to the influence from smaller dimensions of the cylinder discussed in [44] and the later date of testing as described above. As seen, the cube tests are generally consistent with the cylinder tests and they did not exhibit discrepancies (quality issues) during casting. These, together with the fact that cubes had the same age with anchor specimens at testing, are also the reasons for selecting the normalization of all test results as discussed in the previous paragraph based on the measured cube strength. In all cases, the variation of uncracked tests is in the same range of the ones for material characterization tests and overall well within the variation expected in tensile properties of concrete (refer to [45]).

### 3.2. Failure Loads for Uncracked and Cracked Concrete

**Figure 7:**
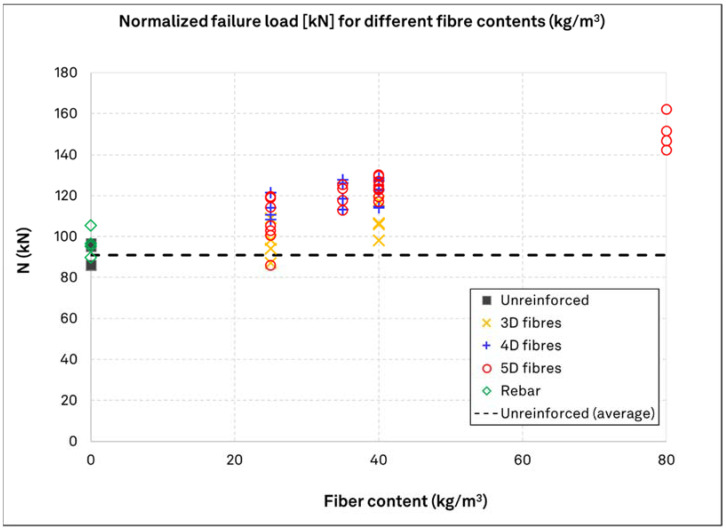
Overall presentation of test results in uncracked concrete, in relation to the fiber type and dosage. The load values are normalized to a 45 MPa cube compressive strength. The dashed line denotes the average resistance in unreinforced concrete, for ease of comparison.

Figure 7 graphically represents the normalized with respect to 45 MPa cube compressive strength results for uncracked concrete in relation to the fiber content and type, and it also indicates the reference strength level, which is the mean resistance of the unreinforced concrete specimens. The tests reinforced with typical rebar are also included in the diagram to compare the influence from the rebar and fibers. As seen, there is a clear overall increase of the concrete breakout load with an increase in the fiber content, although some of the results for a fiber dosage of 25 kg/m^3^ still remain close to reference values. The results from the rebar reinforcement concrete also indicate a minor increase in the load bearing capacity of the anchors at a comparable increment, with that of 3D fibers at 25 kg/m^3^. Moreover, it is shown that the fiber type also strongly influences the performance of the anchors, since tests in concrete with 3D fibers almost consistently exhibit a lower normalized resistance to breakout as compared to concrete with the higher-performance 4D and 5D fibers. As seen, there is a substantial increase in the mean expected load by the inclusion of steel fiber reinforcement, reaching nearly 70% for TS–5D–80–00–60. The efficiency of 4D and 5D fibers as compared to 3D fiber reinforcement is also clearly evident for all cases, excluding TS–5D–25–00–60. At the same time, the contribution of fiber reinforcement to the anchor capacity is very similar for both types of 4D and 5D fibers.

**Figure 8:**
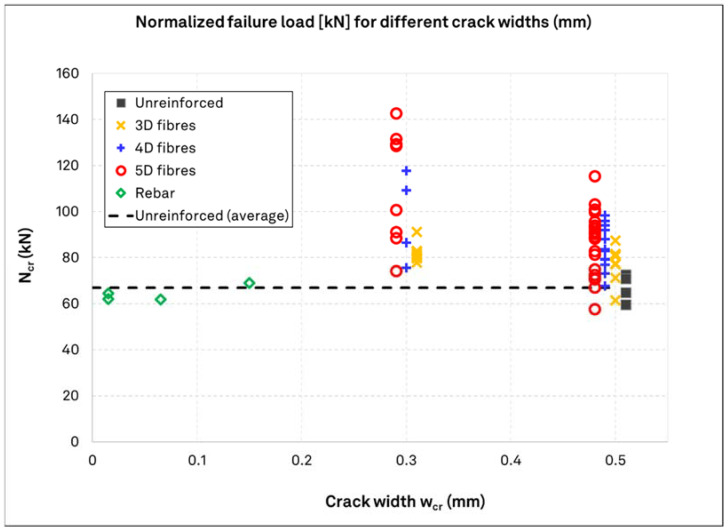
Test results in cracked concrete, in relation to crack width. The reinforcement type (fiber types, rebar reinforcement, unreinforced concrete) is also indicated. The load values are normalized to a 45 MPa cube compressive strength. The dashed line denotes the average resistance in unreinforced concrete, for ease of comparison. Results for crack width 0.3 and 0.5 mm crack widths are offset for presentation reasons.

Figure 8 presents the normalized values of tests in SFRC, unreinforced, and rebar reinforced concrete with different crack widths. Additionally, the average resistance level from the unreinforced concrete is presented here as a reference. For clarity of presentation, the values for crack widths (w_cr_) of 0.3 and 0.5 mm are clustered to different reinforcement types and offset in the graph. Furthermore, the results of the rebar reinforcement configurations have not reached the desired crack width of 0.5 mm with the wedge system used in the tests. Instead, the crack width in this case only ranged between 0.015 mm and 0.15 mm. By comparison of the SFRC values with the reference strength, it becomes evident that the crack width and the fiber type pose a certain influence on the performance of anchors in cracked concrete. As with uncracked concrete, the results for 3D fibers lead to a minor increase to the performance of the anchors. In SFRC, wider cracks lead to lower anchor failure loads. However, the values in cracked SFRC remain, in their majority, higher than in cracked unreinforced concrete. The results from the rebar reinforced concrete have a very different behavior in relation with the crack width. As seen, the anchor performance immediately reduces even for very small crack widths (0.015 mm) and reaches that of the reference tests.

**Figure 9:**
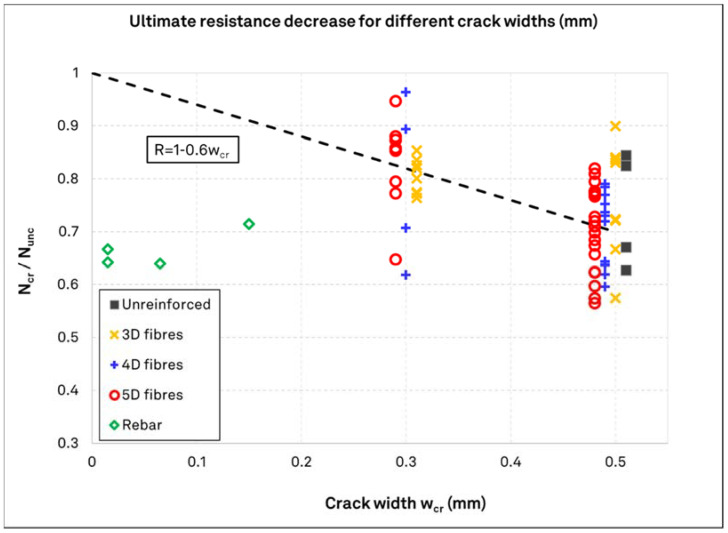
Ratios of normalized anchor resistance in fiber or rebar reinforced concrete over plain concrete with their respective mean resistance and best-fit load reduction function (R = 1–0.6w_cr_) for SFRC test results.

Figure 9 shows the normalized results ratio to their respective uncracked performance. Here, the relation between the crack width and the load reduction becomes more evident. All fiber types indicate a mean load reduction of roughly 20% for a crack width of 0.3 mm and around 30% for a crack width of 0.5 mm. This reduction is also seen for plain concrete with crack width of 0.5 mm, which is in line with the existing literature. The results follow a function of load reduction factor R = 1–0.6·w_cr_, with w_cr_ being the crack width, which can describe the mean load reduction with respect to the crack width for the results presented herein. In this case, a decisive influence of the fiber type is not present. For the rebar reinforced concrete, the reduction in the load bearing capacity of the anchors is approximately 30%, regardless of the crack width. It is also shown that anchors with surface rebar reinforcement do not follow the reduction pattern of the anchors on SFRC. 

### 3.3. Load-Displacement Performance

**Figure 10:**
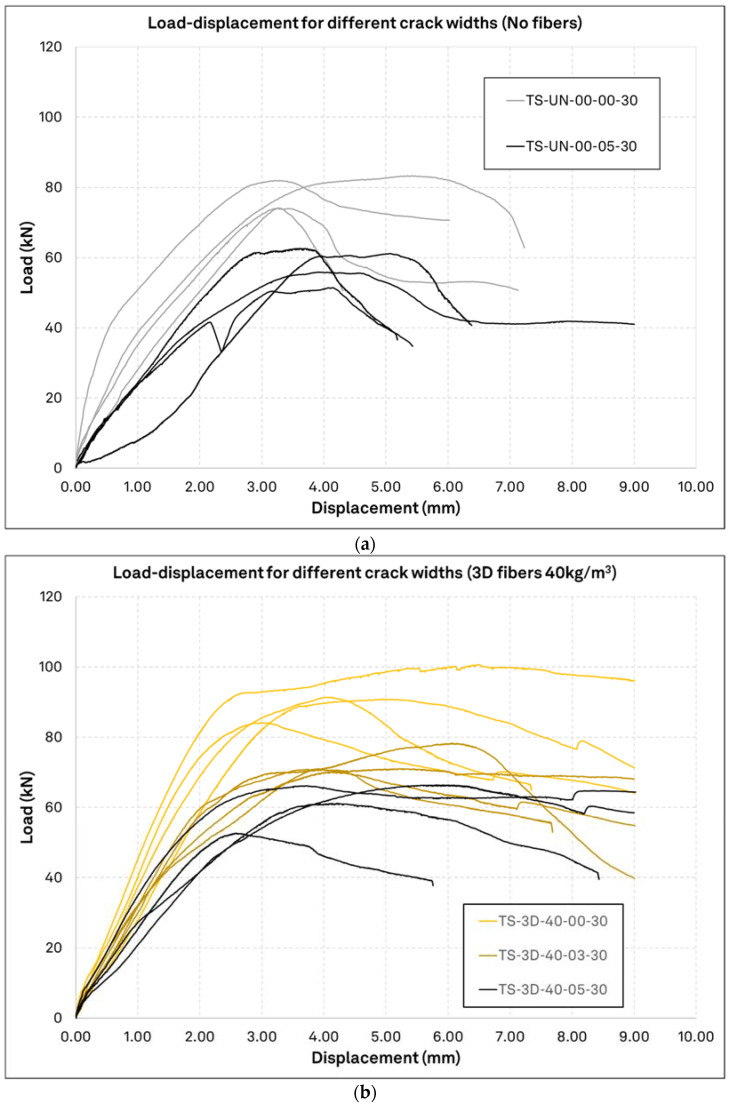
Load displacement curves for different crack widths. Anchors tested in the same concrete specimen with different crack width configuration are plotted together; (**a**): Unreinforced concrete *f_c_* = 33.5 MPa; (**b**): Fiber reinforced concrete 40 kg/m^3^ 3D fibers *f_c_* = 33.2 MPa.

In Figure 10, the load displacement curves from the unreinforced concrete specimens (a) and from the ones with 40 kg/m^3^ of 3D fiber reinforcement (b) are exemplarily presented. The overall influence of the fiber reinforcement on the performance of single anchors can be discerned. The results here are not normalized since all curves in each graph come from the same concrete specimen. The cube compressive strength is almost equal for the unreinforced specimen and the fiber reinforced specimen, with 33.5 MPa and 33.2 MPa, respectively. In the upper graph, the results of both uncracked and cracked with 0.5 mm crack are overlaid and, in the lower graph, this is the case for results of uncracked concrete and crack widths of 0.3 mm and 0.5 mm. Comparing the two figures, it can be seen that fiber reinforcement increased the load bearing capacity and the ductility of the anchors. The initial stiffness of the system is in the same order of magnitude for all anchors, although it gradually decreases with crack opening. Particularly for the fiber reinforced specimens, the load displacement development remains consistently in the linear elastic region for at least 70% of the peak load, whereas unreinforced elements exhibit instabilities earlier on in the load progression. All unreinforced specimens fail at a displacement in the spectrum of 2.0–4.0 mm, but a large number of tests in fiber reinforced specimens exceed this range and, in some cases, reach displacements of 6.0 mm. By observing the load displacement curves, it is evident that the nonlinear behavior of the anchors for cracked and uncracked concrete initiates in the domain of 60 to 65% of the peak load. Finally, the fiber reinforced concrete probes show a slightly higher ductility (area below the load-displacement curve) compared to the unreinforced probes, while in some fiber reinforced probes, a firmly ductile behavior is observed through several cases of nearly flat post-peak load retention. 

**Figure 11:**
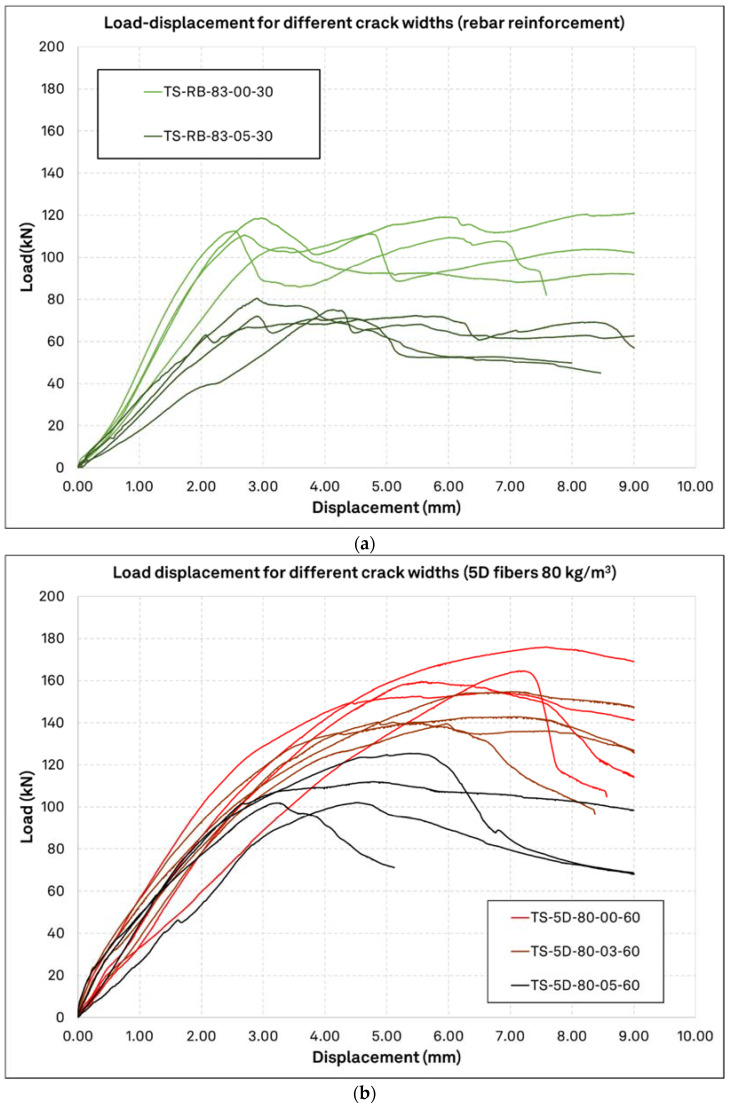
Load displacement curves for different reinforcement configurations and crack widths. Anchors tested in the same concrete specimen with different crack width configuration are plotted together; (**a**): rebar reinforced concrete *f_c_* = 61.3 MPa; (**b**): fiber reinforced concrete 80 kg/m^3^ 5D fibers *f_c_* = 53.1 MPa.

Figure 11a includes the load displacement curves of the single anchors tested in rebar reinforcement for both uncracked and cracked concrete, where the specimen’s cube compressive strength is *f_c_* = 61.3 MPa. In Figure 11b, the single anchor load-displacement curves for cracked concrete with crack width 0.5 mm and 0.3 mm as well as the uncracked concrete results are given for contrast. The concrete in this case is reinforced with 80 kg/m^3^ of 5D fibers, i.e., the configuration with the highest fiber content, and the cube concrete compressive strength is in 53.1 MPa. As shown in the upper graph, the surface reinforcement has increased the ductility significantly in both cracked and uncracked concrete, through activation of the compressive struts in concrete and tensile force retention in the reinforcement, which restrained the development of the concrete breakout body. For the same reason, the aimed crack width was not achieved in the rebar reinforced configuration, and the crack ranged between just 0.015 mm and 0.15 mm instead. However, the resulting load displacement curves barely indicate a difference in ultimate load or ductility, depending on the crack widths. On the other side, it is evident in the bottom graph that the use of 5D fibers with the same total material weight as the rebar have increased the load bearing capacity remarkably and with a certain ductility. The initial stiffness of the anchors in rebar reinforced concrete decreases with crack opening, similarly to unreinforced concrete, as discussed in the previous paragraph, but this is not the case for the 5D fiber reinforced concrete, where a clear distinction of initial anchor stiffness subject to crack opening cannot be identified. Furthermore, the elastic region of the load displacement curve in the concrete with 5D fibers is maintained for a load level of up to 50% of the peak load, which is proportionally smaller as compared to the 3D fiber specimen presented above. Finally, the fiber reinforced concrete probes show some ductility (area below the load-displacement curve) compared to the unreinforced probes, while in some fiber reinforced probes a firmly ductile behavior is observed through a nearly horizontal post-peak load-displacement retention. 

### 3.4. Comparison to Currently Proposed Models

**Figure 12:**
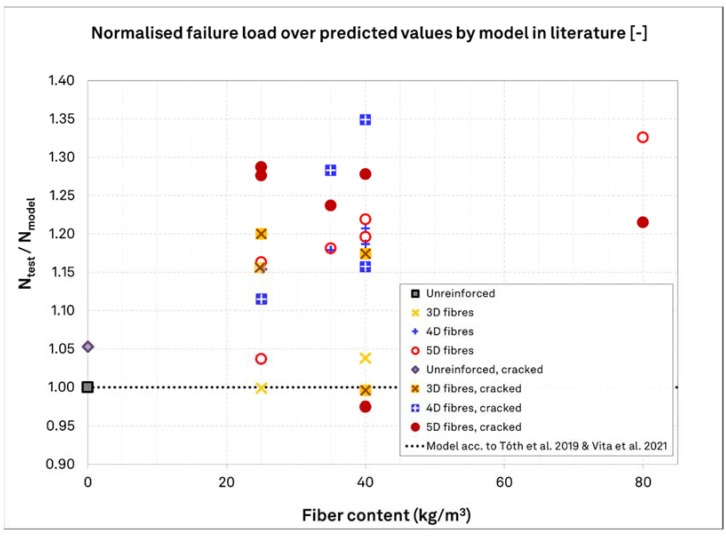
Ratios of normalized values of tests in unreinforced concrete and SFRC over the values estimated by use of the recommendations in [38] for uncracked concrete (Equation (3), accounting for the respective applicability limitations) and in the combination of [35,38] (multiplier of 0.7) for cracked concrete.

Figure 12 presents the ratios of the mean normalized values of tests in unreinforced concrete and SFRC over the values estimated by use of the recommendations in [38] for uncracked concrete (Equation (3)) and in the combination of [35,38] (multiplier of 0.7) for cracked concrete. The reference resistance is not calculated by Equation (1) but it is taken directly from the normalized reference values of TS–Un–00–00–30. For simplicity of presentation, the graph presents the results in perspective of the fiber dosage and dosage, but it does not distinguish cracked widths (for this, it has to be read in conjunction with Table 3). As seen, in most cases the currently proposed design concept underpredict the test results by up to 35% but it captures the test results with adequate safety for some of the test series, namely TS–3D–25–00–30, TS–3D–40–00–30, TS–3D–40–05–30, TS–5D–25–00–60, TS–5D–25–05–60, the latter two being related to uncertain concrete production quality. Consequently, it is evident that the proposed design approach provides a reasonable prediction for 3D fibers in uncracked concrete but in most cases, it is conservative for the case of 3D fibers in uncracked concrete and for 4D and 5D in both uncracked and cracked concrete.

## 4. Conclusions

This study reveals new knowledge in regard to the performance of SFRC structures and, in particular, to the load-bearing performance of single anchors in uncracked and cracked SFRC. Although some studies have been presented in the past and a design proposal is being developed, previous research has not comparatively examined the influence of fibers with different performance characteristics, neither has it posed applications in SFRC in comparison with applications of equivalent rebar reinforcement. The experimental investigations presented herein cover cast-in place headed anchors with varying fiber types, dosages, concrete strengths, and crack widths. Deriving from these studies, the following conclusions can be drawn:

- The addition of fibers clearly leads to increase in the load-bearing resistance at mean values. This increase begins with 10% for 3D fibers at a dosage of 25 kg/m^3^ and goes up to approximately 70% for 5D fibers at a dosage of 80 kg/m^3^, with variation coefficients in a commonplace order of magnitude for concrete related failure modes of fastenings. Additionally, 4D and 5D fibers provide a similar rate of increase. While 3D fibers can still contribute to the concrete breakout capacity, it is to a lesser degree for otherwise the same fiber dosage and concrete strength. Therefore, for the configurations tested applying the same anchor design approach for SFRC as for concrete without fibers is on the safe side.
- The fiber dosage is shown to be a very influencing fiber-related characteristic for the fastening resistance, which agrees with most previous research investigations discussed in the introduction. The type of fibers used has also a very strong influence on the anchor load-bearing resistance. Comparing fibers with hooked-ends and the same length and diameter, but with different material strength and end-anchorage shape, the load increase by use of 3D fibers was in the range of 5 and 15%, while 4D and 5D fibers led to an overall resistance increase in the range of 10 and 40% for fiber dosages of 25 kg/m^3^ and 40 kg/m^3^, respectively.
- Fiber reinforced concrete has indicated a beneficial behavior in cracked concrete. The load bearing capacity of anchors in cracked fiber reinforced concrete was higher than in unreinforced concrete. A relation of the fiber type and the anchor performance in cracked concrete is evident. The 3D fibers have the least influence, while 4D and 5D fiber have a similar effect. Considering solely the SFRC specimens tested herein, the mean load reduction of anchors in cracked SFRC in relation to the crack width can be described by an analogy to the crack width, which is in reasonable agreement with the few previous studies discussed in the introduction.
- The surface rebar reinforcement does not influence the load bearing capacity significantly. The resistance increment for a favorably arranged reinforcement presented herein is similar to that of 3D fibers with 25 kg/m^3^. There is no contribution of the rebar to the load capacity in cracked concrete, and there is no apparent correlation between the crack width and the load reduction. Nonetheless, in both cracked and uncracked concrete the ductility of the anchors has increased remarkably.
- Both typical surface reinforcement and fiber reinforcement in concrete influence the ductility of single anchors. There is notable increase in the ductility of the anchors behavior in the case of concrete cone failure mode for both types of reinforcement. For the same overall steel reinforcement quantity, high performance fibers provide a significantly higher load bearing capacity over that of rebar reinforcement.
- The adequacy of currently proposed design approaches in [33,35,38] is confirmed by the tests herein. In particular, the linear increase in load-bearing capacity by increasing fiber content as proposed by the equation in [33,38] is a reasonable principle. This equation can predict the results with a reasonable accuracy for 3D fibers and with conservatism for 4D and 5D fibers. The test results confirm that the equation is applicable for a ratio of fiber length to embedment of 1.7. Furthermore, the high scatters in test results for concrete with fiber reinforcement at 25 kg/m^3^ advocates for an applicability limit at or close to this lower bound fiber dosage. The reduction multiplier of 0.7 for the prediction of the load capacity in concrete anchors located in a 0.3 mm crack, as proposed by [35], is in agreement with the investigation results.

## Figures and Tables

**Table 1:** Overview of test program.

Test Series	Reinforcement Type	Fiber Dosage (kg/m^3^)	Crack Width (mm)	Target Concrete Strength (MPa)
TS-UN-00-00-30	-	-	-	30
TS-UN-00-05-30	-	-	0.5	30
TS-3D-25-00-30	3D	25	-	30
TS-3D-25-03-30	3D	25	0.3	30
TS-3D-25-05-30	3D	25	0.5	30
TS-3D-40-00-30	3D	40	-	30
TS-3D-40-03-30	3D	40	0.3	30
TS-3D-40-05-30	3D	40	0.5	30
TS-4D-25-00-30	4D	25	-	30
TS-4D-25-05-30	4D	25	0.5	30
TS-4D-40-00-30	4D	40	-	30
TS-4D-40-05-30	4D	40	0.5	30
TS-4D-40-00-60	4D	40	-	60
TS-4D-40-03-60	4D	40	0.3	60
TS-4D-40-05-60	4D	40	0.5	60
TS-4D-35-00-40	4D	35	-	40
TS-5D-25-00-60	5D	25	-	60
TS-5D-25-05-60	5D	25	0.5	60
TS-5D-25-00-30	5D	25	-	30
TS-5D-25-03-30	5D	25	0.3	30
TS-5D-25-05-30	5D	25	0.5	30
TS-5D-35-00-40	5D	35	-	40
TS-5D-40-00-40	5D	40	-	40
TS-5D-40-05-40	5D	40	0.5	40
TS-5D-40-00-60	5D	40	-	60
TS-5D-40-05-60	5D	40	0.5	60
TS-5D-80-00-60	5D	80	-	60
TS-5D-80-03-60	5D	80	0.3	60
TS-5D-80-05-60	5D	80	0.5	60
TS-RB-83-00-60	RB	83	-	30
TS-RB-83-05-60	RB	83	0.5	30

**Table 2:** Properties of fibers used.

Product Type	Diameter [mm]	Length [mm]	Nominal Tensile Strength [MPa]
Dramix 3D	0.90	60	1160
Dramix 4D	0.90	60	1500
Dramix 5D	0.90	60	2300

**Table 3:** Summary of test results.

Test Set ID	Mean Failure Load [kN]	Mean Failure Load Normalised to 45 Mpa [MPa]	Coeff. of Variation of Failure Load [—]	Mean Cube Compressive Strength [MPa]	Mean Cylinder Compressive Strength [MPa]
TS-3D-25-00-30	77.05	98.39	0.094	28.33	29.850
TS-3D-25-03-30	63.23	79.68	0.014		
TS-3D-25-05-30	65.64	82.72	0.038		
TS-3D-40-00-30	91.74	106.96	0.074	33.1	33.270
TS-3D-40-03-30	72.56	84.61	0.053		
TS-3D-40-05-30	61.60	71.82	0.104		
TS-4D-25-00-30	87.54	113.62	0.051	26.71	35.03
TS-4D-25-05-30	59.22	76.87	0.101		
TS-4D-40-00-30	93.06	124.36	0.056	25.2	31.95
TS-4D-40-05-30	62.44	83.44	0.119		
TS-4D-40-00-60	141.45	122.27	0.047	60.22	58.10
TS-4D-40-03-60	112.51	97.25	0.202		
TS-4D-40-05-60	107.00	92.49	0.037		
TS-4D-35-00-40	99.81	121.43	0.056	30.4	30.4
TS-5D-25-00-30	94.25	114.55	0.059	30.46	35.1
TS-5D-25-03-30	72.97	88.69	0.124		
TS-5D-25-05-30	72.33	87.91	0.054		
TS-5D-25-00-60	118.75	102.10	0.133	60.87	52.26
TS-5D-25-05-60	78.12	67.17	0.109		
TS-5D-35-00-40	107.01	119.83	0.047	35.883	32.25
TS-5D-40-00-40	107.18	123.21	0.026	34.05	35.24
TS-5D-40-05-40	76.21	87.61	0.155		
TS-5D-40-00-60	147.19	125.53	0.048	61.87	55.73
TS-5D-40-05-60	104.55	89.16	0.116		
TS-5D-80-00-60	163.58	150.65	0.056	53.06	56.25
TS-5D-80-03-60	144.47	133.04	0.049		
TS-5D-80-05-60	110.36	101.64	0.101		
TS-RB-83-00-60	112.86	96.72	0.067	61.27	55.68
TS-RB-83-05-60	76.31	65.39	0.078		
TS-Un-00-00-30	78.39	90.88	0.063	33.48	37.86
TS-Un-00-05-30	57.77	66.97	0.089		

## Data Availability

Data available on request.
